# Bicervical Normal Uterus with Normal Vagina and Anteroposterior Disposition of the Double Cervix

**DOI:** 10.1155/2011/303828

**Published:** 2011-07-07

**Authors:** José Morales-Roselló, Núria Peralta LLorens

**Affiliations:** Clínica Morales, C/Trafalgar 46 10 2a, 46023 Valencia, Spain

## Abstract

We report a very uncommon uterine anomaly consisting on a normal uterus, a double cervix with an anteroposterior disposition, and absence of vaginal septum. A 36-years-old woman with one child and absence of past reproductive disorders was examined for a routine checkup. Clinical and transvaginal ultrasound examinations showed a normal uterus with a double cervix disposed in an anteroposterior fashion with the absence of vaginal septum. A review of the theories concerning müllerian fusion is done, and implications of this case in relation with these theories are discussed. This is the first case of a normal uterus with a double cervix situated in an anteroposterior fashion and absence of vaginal septum. This case is in concordance with theories that consider the fusion of the caudal part of Müllerian ducts to be the result of a complex process. It proves that at least in some cases the most caudal part of müllerian ducts is fused in an anteroposterior disposition.

## 1. Introduction

Embryology of the uterus is an area of anatomical research that has long ago been considered as concluded as most of the anomalies do follow a pattern of complete or partial absence of Müllerian fusion. Little research has been done trying to understand the physiopathology of anomalies falling outside the common accepted explanation. 

This case supports those investigators who think there are still unanswered questions concerning the embryology of reproductive organs and is in concordance with some of the theories put recently on the scientific scenario.

## 2. Case Report

A 36-year-old woman with past medical history of menarche at the age of 13, one vaginal delivery at term, and absence of miscarriages or reproductive disorders was attended in our private clinic for a routine checkup. 

**Figure 1:**
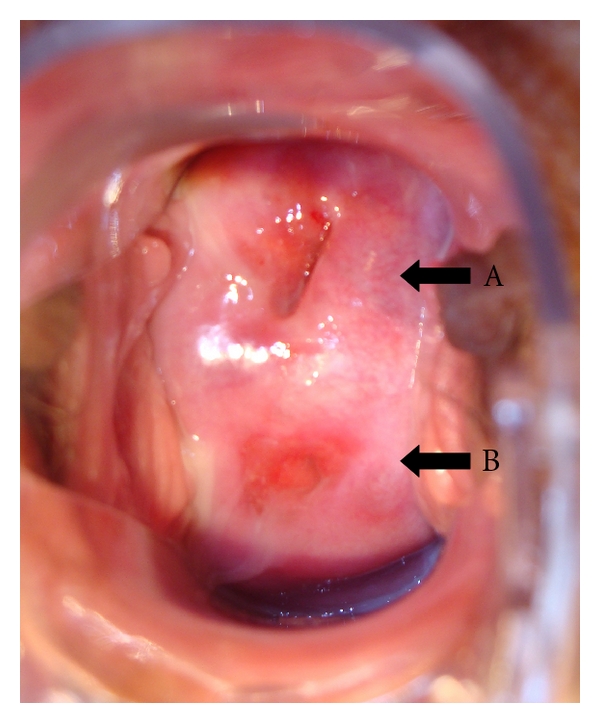
Picture of the dual cervix (black arrows A, B). Both cervices are situated in an anteroposterior disposition. A reddish endocervical epithelium can be clearly seen surrounding both external os.

Examination of the vagina showed a double cervix with a normal vagina without any septum. Interestingly, both cervices were situated in an anteroposterior disposition with the posterior one slightly less developed (Figure 1). In both cervices, an evident external os surrounded with a reddish endocervical epithelium was present. 

**Figure 2:**
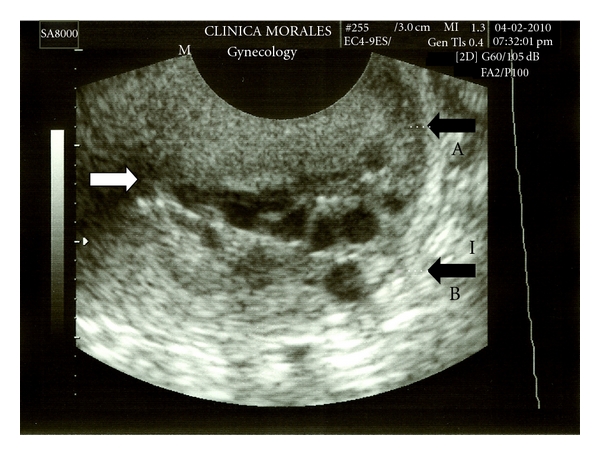
Transvaginal ultrasound depicts an axial section of the uterus; a normal anteroflexed uterus is seen with a double cervix (black arrows A, B). Both endocervical channels join together. White arrow points to the place at the internal cervical os where the channels fuse.

Transvaginal ultrasound clearly showed a dual cervix with a normal anteroflexed uterus (Figure 2). Both endocervical channels were joined together in a common internal os, and both were surrounded throughout their ascent by small mucous cysts, typical of the functional endocervical crypts, as a normal endocervix has no proper endocervical glands. The ovaries and the pouch of Douglas were also normal.

Unfortunately, as the patient was clinically asymptomatic, she refused to have more examinations performed, so we could not obtain permission for a hysterosalpingography or an intravenous urogram. However, we considered that hysterosalpingography would not yield much information. As both endocervical channels were joined together in a common channel, uterine cavity and fallopian tubes would not differ from normality.

## 3. Discussion

This is the first report of a normal uterus with a cervical duplication and a normal vagina. Previous reports always included a vaginal septum [1–5]. Also, this is the first report of a normal uterus with a cervical duplication and an anteroposterior disposition of this double cervix. 

Classically, congenital uterine anomalies have been associated with reproductive complications (sterility, early pregnancy loss, and preterm delivery). Of these, the uterine septum is the most frequent anomaly and the most related with failure of implantation [6, 7]. Although some studies report bicornuate uterus as the congenital anomaly with poorer outcome [8], this, as well as the unicornuate uterus, is more prone to cause midtrimester pregnancy loss and preterm delivery later in gestation [6].

These anomalies have been classically described according to the American Fertility Society classification. This classification was based on anatomical research that correlated anomalies with the fusion of the Müller ducts: Jarko (1946), Crosby and Hill (1962), and others. They assumed that these paramesonephric structures fuse in an upward fashion: caudal to cranial. According to this scheme, anomalies occur when Müllerian fusion does not progress over a certain point. Also, in the vagina, malformation may occur when tissue resorption is incomplete.

However, many reports exist without concordance with this hypothesis. The work of Müller in 1967 stated that the tubes fusion is achieved in a bidirectional fashion: cranial to caudal and caudal to cranial with a starting point close to the isthmus. Although this explains many other cases, it is still incomplete and does not explain the physiopathology of complex malformations such as the congenital blind hemivagina with renal hypoplasia and the unilateral renal hypoplasia with ureteral anomalies. 

An interesting hypothesis (clinical and embryological) formulated by Acien, based on microscopic analysis of the mesonephric (wolffian) and paramesonephric (Müllerian) tubes of the rat, considered that the origin of the vagina is derived from the wolffian ducts together with the Müller tubercle. As the ureteral sprout has a wolffian origin, this explains the association of vaginal anomalies and urologic hemihypoplasias or hemiaplasias [9]. 

Also, according to anatomical data [10, 11], the Müllerian tubes fuse and again separate at the most caudal part and can be differentiated according to this special fusion into three distinct segments: one segment (the one fused cranially) forms the uterus, the second segment (formed by the beginning and the end of the Müllerian ducts' divergence) forms the internal cervical os and the external cervical os, and the third and most caudal segment is the one in which the tubes converge with the vaginal sinus forming the Müllerian tubercle. This last structure forms the vagina in association with the cells of wolffian origin, therefore making the vagina an organ with a mesonephric (wolffian) and paramesonephric (Müllerian) origin.

The case presented agrees with Acien's theory as (1) the defect starts in the internal cervical os and extends reaching the external cervical os, (2) no anomalies were found in the uterus showing that the upper part of the fusion was properly completed, and (3) the lower part of the fusion was also correctly completed with a consequent normal vagina. 

Interestingly, the disposition of the cervix was seen in an anteroposterior fashion. This might be due to the fact that the fusion of the Müllerian tubes with the vaginal sinus is not always achieved in a side-by-side position. In some cases, this might be achieved in an anteroposterior plane, which is in concordance with some malformations of the vagina where an anterior and a posterior vagina may be found.

In summary, the mechanisms of fusion of the Müllerian ducts at their caudal end are not as simple as were supposed. This case agrees with Acien's theory and proves the possibility of a different disposition of the ducts' caudal end during this process.

## References

[1] Goldberg JM, Falcone T (1996). Double cervix and vagina with a normal uterus: an unusual Mullerian anomaly. *Human Reproduction*.

[2] Dunn R, Hantes J (2004). Double cervix and vagina with a normal uterus and blind cervical pouch: a rare müllerian anomaly. *Fertility and Sterility*.

[3] Varras M, Akrivis C, Demou A, Kitsiou E, Antoniou N (2007). Double vagina and cervix communicating bilaterally with a single uterine cavity: report of a case with an unusual congenital uterine malformation. *Journal of Reproductive Medicine*.

[4] Pavone ME, King JA, Vlahos N (2006). Septate uterus with cervical duplication and a longitudinal vaginal septum: a müllerian anomaly without a classification. *Fertility and Sterility*.

[5] Shirota K, Fukuoka M, Tsujioka H, Inoue Y, Kawarabayashi T (2009). A normal uterus communicating with a double cervix and the vagina: a müllerian anomaly without any present classification. *Fertility and Sterility*.

[6] Taylor E, Gomel V (2008). The uterus and fertility. *Fertility and Sterility*.

[7] Propst AM, Hill JA (2000). Anatomic factors associated with recurrent pregnancy loss. *Seminars in Reproductive Medicine*.

[8] Acien P (1993). Reproductive performance of women with uterine malformations. *Human Reproduction*.

[9] Acien P (1992). Embryological observations on the female genital tract. *Human Reproduction*.

[10] Sánchez-Ferrer ML, Acién MI, Sánchez del Campo F, Mayol-Belda MJ, Acién P (2006). Experimental contributions to the study of the embryology of the vagina. *Human Reproduction*.

[11] Acién P, Acién M, Sánchez-Ferrer M (2004). Complex malformations of the female genital tract. New types and revision of classification. *Human Reproduction*.

